# Editorial: Reconstitution methods in cell biology

**DOI:** 10.3389/fcell.2026.1980819

**Published:** 2026-10-09

**Authors:** Coral Y. Zhou, Laurent Blanchoin, Jennifer Landino

**Affiliations:** 1 Department of Molecular Biosciences, The University of Kansas, Lawrence, KS, United States; 2 Cytomorpholab, Cell and Plant Physiology Laboratory, Interdisciplinary Research Institute of Grenoble, University of Grenoble-Alpes, CEA, CNRS, INRA, Grenoble, France; 3 Department of Biochemistry and Cell Biology, Geisel School of Medicine, Dartmouth College, Hanover, NH, United States

**Keywords:** cytoskeleton, *in vitro*, membrane, nucleus, reconstitution

## Introduction

Reconstitution uncovers the principles that govern complex cellular behavior. By distilling cells into minimal, tractable systems, cell-free approaches have revealed much of how cells organize and function, exposing the biochemical and biophysical mechanisms behind emergent behavior. But simplification poses the field’s central tension: how do we infer physiological relevance from a reduced system, and reintroduce complexity so insights translate across minimal, semi-defined, and *in vivo* contexts? This Research Topic highlights the interdisciplinary advances now meeting that challenge.

## Confinement

The cytosol of the cell is crowded, heterogeneous, and isolated from the external environment by the plasma membrane. Reconstitution studies are traditionally performed in volumes that far exceed that of a typical cell, and therefore do not mimic the physiology of the cytosol. Recent studies have developed methods to confine protein mixtures or cytosol within oil-in-water droplets or membrane enclosed vesicles as a way to address this limitation. These confinement approaches are technically challenging both to develop and to analyze by microscopy. In Vianay et al. the authors describe methods for generating microwells which impose volume and mechanical constraints on reconstituted cytoskeletal networks. Notably, these microwells can be engineered with a variety of geometries, heights, and volumes, and they can be used with multiple imaging modalities, highlighting the versatility of this approach. The confinement of reconstituted systems into cell-like compartments will be an important tool for future studies on how limiting components drive biological phenomena.

## Mechanics

Reconstitution of the cellular cytoskeleton exemplifies the challenge of balancing molecular simplification and physiological relevance. An actin monomer, a single actin filament, an array of actin filaments, and an actomyosin network all display unique properties and behaviors. While the regulation of filament assembly, disassembly, motor-dependent force generation and transport has been well-characterized for single filaments and their interaction proteins, the large-scale behaviors of cytoskeletal networks are less well understood. In Amari et al. the authors describe quartz crystal microbalance with dissipation monitoring (QCM-D) as a tool for measuring emergent mechanics in actomyosin networks. They demonstrate that QCM-D can measure viscoelasticity in the network under conditions that mimic the physiological complexity of the cell. In Godfrey and Gurmessa, the authors investigate the mesoscale mechanics of cross-linked actin networks. They find that networks of mixed actin crosslinkers provide a mechanism to tune the mechanical properties of the network while simultaneously maintaining connectivity. Both studies demonstrate the shift toward reconstituting complex cytoskeletal networks and measuring emergent properties to define the mechanisms that support cell motility, adhesion, and division.

## Oscillatory systems

For oscillatory systems in particular, the combination of theoretical modeling with experimental *in vitro* studies has been a powerful approach to push our understanding forward. In van den Vlist et al. the authors review the principles that define biological oscillations as well as the diverse array of oscillatory systems that have been examined using reconstitution including the circadian clock, transcription-translation oscillators, and a post-translational oscillator (MinD-MinE). Notably, the authors highlight the challenges of reconstituting oscillatory systems, including the difficulty of identifying the narrow parameter space where oscillations can occur. In this way, *in vitro* reconstitution represents a double-edged sword. The lack of successful reconstitution may indicate either a failed experiment or a “hidden” component required for the phenomenon to emerge. Identifying additional complexities or dependencies through efforts to reconstitute biological oscillations can bridge the gap between simplified mathematical models and noisy, heterogeneous, cellular behaviors.

## Multi-component complexes

While the tools for isolating and studying single proteins *in vitro* have been well established, the toolkit for multi-protein complexes is more limited. One way to investigate processes controlled by large complexes is through semi-defined reconstitution from cell lysates rather than *de novo* assembly from purified proteins. This approach overcomes the technical challenge of optimizing expression and purification for each component of the complex and takes advantage of the fact that biology has already solved this problem. Larson et al. describe how yeast extracts can be used to reconstitute kinetochore assembly and microtubule capture. As yeast are a genetically tractable system, this approach allows investigators to integrate endogenous genetic manipulation (tagging, deletion, mutagenesis) with perturbations only feasible in an “open” biochemical system (oscillating flow). Notably, this method allows for the study of a multi-protein complex isolated from the surrounding cytosol at the single molecule level.

## Membrane fusion

The strength of cell-free reconstitution is not limited to the study of protein-only assemblies, it is also a powerful system for understanding biological membranes. Membrane fusion is a dynamic, energetically unfavorable event that depends on a complex network of protein-protein and protein-lipid interactions. Authors Madison and Yokom review the advancements in our understanding of membrane fusion gained through *in vitro* reconstitution studies. This work spans from large-scale bulk fusion assays down to single fusion events observed through a combination of cryo-electron tomography to resolve spatial organization and total internal reflection fluorescence microscopy to quantify fusion dynamics. The authors highlight how these tools will advance the field’s understanding in new areas, for example, in asymmetric fusion events.

## Nuclear biology

The nucleus is the largest and most easily identifiable organelle in typical eukaryotic cells and aberrant nuclear structure has long been understood to be a hallmark of diseases like cancer. Sengupta et al. review the key understanding of regulators of nuclear morphology in health and disease states, including knowledge gained from studies performed in the *Xenopus* egg extract system which support physiological nuclear assembly. Reconstitution of nuclei *in vitro* provides a high-throughput system for investigating the role of nuclear lamina, cytoskeleton, nucleocytoplasmic transport, and chromatin and epigenetic factors in regulating nuclear architecture. The authors particularly emphasize the emergence of chromatin and nuclear structural proteins and key regulators of nuclear structure and function, and highlight the field’s move toward interface of chromatin biology, nuclear morphology, and cell health. Lenz and Zhou, reviews additional approaches that use *Xenopus* egg extracts to visualize a wide array of genome remodeling events including chromatin assembly, DNA replication and repair, and mitotic chromosome formation. Combining these classic assays with advanced techniques in imaging and biochemistry have revealed new insight into the biochemical regulation of these key chromatin remodeling events. Finally, the authors speculate how the *Xenopus* egg extract system can be used in the future for discovering the molecular basis of genome evolution and speciation.

## Conclusion

Cell-free reconstitution is an iterative methodology that balances two fundamental questions. 1) what is the minimal set of components that can produce this behavior? 2) how do complex phenomena emerge from the interaction of individual molecules? Emerging interdisciplinary approaches now enable the engineering of physiologically relevant reconstituted systems to probe cell function and uncover general principles of living systems.

